# BRAZILIAN HERNIA AND ABDOMINAL WALL SOCIETY STATEMENT ON LARGE HIATAL HERNIAS MANAGEMENT

**DOI:** 10.1590/0102-672020230069e1787

**Published:** 2024-02-05

**Authors:** André BRANDALISE, Fernando Augusto Mardiros HERBELLA, Renato Abrantes LUNA, Sergio SZACHNOWICZ, Rubens Antonio Aissar SALLUM, Carlos Eduardo DOMENE, Paula VOLPE, Leandro Totti CAVAZZOLLA, Marcelo Lopes FURTADO, Christiano Marlo Paggi CLAUS, José Francisco de Mattos FARAH, Eduardo CREMA

**Affiliations:** 1 Centro Médico de Campinas, Robotic Surgery Program – Campinas (SP), Brazil;; 2 Universidade Federal de São Paulo, Department of Surgery, – São Paulo (SP), Brazil;; 3 Universidade Federal do Estado do Rio de Janeiro, Faculty of Medicine and Surgery, Hospital Federal dos Servidores do Estado – Rio de Janeiro (RJ), Brazil;; 4 Universidade de São Paulo, Department of Gastroenterology – São Paulo (SP), Brazil;; 5 Sociedade Brasileira de Cirurgia Laparoscópica e Robótica – São Paulo (SP), Brazil;; 6 Centro Integrado de Medicina Avançada – São Paulo (SP), Brazil;; 7 Universidade Federal do Rio Grande do Sul, University Hospital, General Surgery Unit – Porto Alegre (RS), Brazil;; 8 Sociedade Brasileira de Hérna e Parede Abdominal, Hospital Pintagueiras, Videolaparoscopic Surgery Service – Jundiaí, São Paulo (SP), Brazil;; 9 Universidade Positivo, Postgraduate Program in Minimally Invasive Surgery, Department of Surgical Clinic – Curitiba (PR), Brazil;; 10 Hospital do Servidor Público Estadual, Department of General and Oncological Surgery – São Paulo (SP), Brazil;; 11 Universidade Federal do Triângulo Mineiro, Digestive Surgical Unit – Uberaba (MG), Brazil.

**Keywords:** Hiatal Hernia, General Surgery, Fundoplication, Laparoscopy, Robotic Surgery, Surgical Mesh, Hérnia Hiatal, Cirurgia Geral, Fundoplicatura, Laparoscopia, Cirurgia Robótica, Telas Cirúrgicas

## Abstract

Large hiatal hernias, besides being more prevalent in the elderly, have a different clinical presentation: less reflux, more mechanical symptoms, and a greater possibility of acute, life-threatening complications such as gastric volvulus, ischemia, and visceral mediastinal perforation. Thus, surgical indications are distinct from gastroesophageal reflux disease-related sliding hiatal hernias. Heartburn tends to be less intense, while symptoms of chest pain, cough, discomfort, and tiredness are reported more frequently. Complaints of vomiting and dysphagia may suggest the presence of associated gastric volvulus. Signs of iron deficiency and anemia are found. Surgical indication is still controversial and was previously based on high mortality reported in emergency surgeries for gastric volvulus. Postoperative mortality is especially related to three factors: body mass index above 35, age over 70 years, and the presence of comorbidities. Minimally invasive elective surgery should be offered to symptomatic individuals with good or reasonable performance status, regardless of age group. In asymptomatic and oligosymptomatic patients, besides obviously identifying the patient’s desire, a case-by-case analysis of surgical risk factors such as age, obesity, and comorbidities should be taken into consideration. Attention should also be paid to situations with greater technical difficulty and risks of acute migration due to increased abdominal pressure (abdominoplasty, manual labor, spastic diseases). Technical alternatives such as partial fundoplication and anterior gastropexy can be considered. We emphasize the importance of performing surgical procedures in cases of large hiatal hernias in high-volume centers, with experienced surgeons.

## RESUMO

As grandes hérnias de hiato, além de serem mais prevalentes em idosos, têm apresentação clínica diferente: menos refluxo, mais sintomas mecânicos e maior possibilidade de complicações agudas e potencialmente fatais, como vólvulo gástrico, isquemia e perfuração mediastinal visceral. Assim, as indicações cirúrgicas são distintas das hérnias de hiato por deslizamento, relacionadas à doença do refluxo gastroesofágico. A azia tende a ser menos intensa, enquanto os sintomas de dor no peito, tosse, desconforto e cansaço são relatados com maior frequência. Queixas de vômitos e disfagia podem sugerir a presença de volvo gástrico associado. São encontrados sinais de deficiência de ferro e anemia. A indicação cirúrgica ainda é controversa e foi anteriormente baseada na alta mortalidade relatada em cirurgias de emergência para volvo gástrico. A mortalidade pós-operatória está especialmente relacionada a três fatores: índice de massa corporal acima de 35, idade superior a 70 anos e presença de comorbidades. A cirurgia eletiva minimamente invasiva deve ser oferecida a indivíduos sintomáticos, com desempenho bom ou razoável, independentemente da faixa etária. Em pacientes assintomáticos e oligossintomáticos, além de obviamente identificar o desejo do paciente, deve-se levar em consideração a análise caso a caso dos fatores de risco cirúrgico, como idade, obesidade e comorbidades. Deve-se atentar também para situações de maior dificuldade técnica e riscos de migração aguda por aumento da pressão abdominal (abdominoplastia, trabalhos manuais, doenças espásticas). Alternativas técnicas como fundoplicatura parcial e gastropexia anterior podem ser consideradas. Ressaltamos a importância da realização de procedimentos cirúrgicos nos casos de grandes hérnias de hiato em centros de grande volume, com cirurgiões experientes.

## INTRODUCTION

Large hiatal hernias (LHH) management, also known as intrathoracic stomach or giant hiatal hernias, is a controversial topic.

Hiatal hernias may be classified as:

Type I – sliding;

Type II – paraesophageal hernia;

Type III – mixed sliding and paraesophageal; and

Type IV – associated with the herniation of other organs^29^.

The Brazilian Hernia and Abdominal Wall Society held a consensus meeting on the area based on the experience of national expert surgeons and current literature.

We arranged the main topics regarding the management of LHH in questions that were presented by two experts and discussed among the others until a consensus was reached.

## DEFINITION

The group considered for this statement were only LHH types II, III, and IV, defined as those with more than 5 cm or one-third of herniated stomach into the mediastinum.

## ROUTINE SURGICAL THERAPY FOR ASYMPTOMATIC OR OLIGOSYMPTOMATIC PATIENTS

- LHH are most commonly found after the seventh decade of life.
- The vast majority is asymptomatic, and the chances of acute complications are low.
- When complications occur, especially gastric volvulus, mortality rates are high.

LHH, besides being more prevalent in the elderly, have different clinical presentation: less reflux, more mechanical symptoms, and a greater possibility of acute, life-threatening complications such as gastric volvulus, ischemia, and visceral mediastinal perforation. Thus, surgical indications are distinct from gastroesophageal reflux disease (GERD)-related sliding hiatal hernias^18,20,27^.

Heartburn tends to be less intense, while symptoms of chest pain, cough, discomfort, and tiredness are reported more frequently. Complaints of vomiting and dysphagia may suggest the presence of associated gastric volvulus. Signs of iron deficiency and anemia are found in almost 20% of cases^9^.

Surgical indication in patients with LHH is still controversial and was previously based on high mortality reported in emergency surgeries for gastric volvulus^7,18,28^.

In the 1980s and 1990s, publications with analyses of large populations demonstrated that acute complications, although severe and of high mortality, are less frequent than was once imagined and asymptomatic or oligosymptomatic patients may be followed up without surgical intervention. Watch and wait is an acceptable approach, especially based on the findings that the overall mortality was 5.4% and the frequency of 1.1% per year of asymptomatic cases required surgery^45^. More recent studies highlight the possibility of observation in asymptomatic patients, although surgery can be safely offered and good results may surge in patients with controlled comorbidities regardless of age group^32^.

On the other hand, acute complications such as gastric volvulus with incarceration or vascular suffering remain one of the most dramatic and urgent situations, especially in the elderly^10^. A recent publication compared a series of urgent surgeries in LHH and showed a 1.7-fold increase in morbidity and a 2.7-fold increase in mortality when compared to elective surgery, even after age adjustment and comorbidities^14^. These authors recommend elective and minimally invasive surgery for symptomatic and oligosymptomatic patients considered fit for the procedure. This onset of paradigm shift (contrary to observation in asymptomatic patients) can be considered an effect of the results of minimally invasive surgery, which showed complication and mortality rates lower than 1% in large series^10,18^.

Postoperative mortality is especially related to three factors: body mass index above 35, age over 70 years, and the presence of comorbidity (Charlson index greater than 3)^2^. Age-related mortality in patients under 60 years of age or between 60 and 69 years is 0%, between 70 and 79 is 0.9%, and in patients over 80 years, mortality is 7.8% ^28,29^. In general, mortality between 0.9 and 4.6% is described after elective correction of LHH^13^.

### Recommendations

Minimally invasive elective surgery should be offered to symptomatic individuals with good or reasonable performance status, regardless of age group.

In asymptomatic and oligosymptomatic patients, besides obviously identifying the patient’s desire, a case-by-case analysis of surgical risk factors such as age, obesity, and comorbidities, should be taken into consideration. Attention should also be paid to situations with greater technical difficulty and risks of acute migration due to increased abdominal pressure (abdominoplasty, manual labor, spastic diseases).

We emphasize the importance of performing surgical procedures in cases of LHH in high-volume centers, with experienced surgeons.

## PRE-OPERATIVE WORK-UP

The commonly used work-up in LHH includes: upper digestive endoscopy, barium upper gastrointestinal (GI) series, prolonged esophageal pH monitoring (pH-metry), esophageal manometry, and computed tomography (CT).

### Upper gastrointestinal endoscopy

The upper GI is most commonly performed when the patient’s complaint is reflux, regurgitation, or dysphagia. The examination is challenging in very large hernias, with alteration in gastric anatomy and esophageal tortuosity. Sometimes endoscopy fails to estimate the size of the hernia or the length of the esophagus, and should always be associated with an imaging test^27^.

In emergency cases endoscopy can decompress an obstructed stomach and evaluate the mucosal integrity.

### Upper gastrointestinal barium series

The upper gastrointestinal barium series is a non-invasive and low-cost test that gives an overview of the hernia, which sometimes becomes difficult by endoscopy.

It is possible to identify the size of the hernia, the location of the esophagogastric junction (EGJ) in relation to the diaphragm, and the shape of the herniated stomach, whether anatomical or with some type of volvulus^47^.

Esophageal shortening or stenosis is also very well evaluated. In cases when a motility test is not feasible, it shows, especially when a video barium swallow test is performed, the esophageal emptying.

### Computed tomography scan

Nowadays we have seen many patients referred to a CT scan for the investigation of thoracic symptoms that detects LHH. It allows a good anatomical vision of the hernia and identifies other herniated organs.

CT scan is especially useful in acute cases, where a complication of hiatal hernia, such as volvulus or perforation, is suspected. The use of venous contrast may evaluate perfusion changes in the stomach or other herniated organs.

Due to the ability to make sagittal, coronal, and axial cuts and three-dimensional reconstructions, it is also possible to evaluate the diameter of the hiatal defect at its rest position and determine the herniated volume to try predicting cases of greater complexity^24,26^.

### Esophageal manometry

Many patients with LHH have dysphagia as part of their symptoms, and evaluating esophageal motility is important, not for the diagnosis of the disease, but for the planning of the surgical procedure^15^.

The test itself bares greater technical difficulty since frequently it is not possible to transpose the EGJ with the probe. If this is the case, it can be guided by endoscopy before the examination or only the esophageal body may be studied to assess whether there is adequate motility for a fundoplication.

Although up to 53% of motor alterations are found in patients with LHH, those may be secondary to the hernia and would not have a negative influence on the results of surgeries in relation to patients without motor alterations detected^52^.

Manometry can be conventional or high resolution, and there is no clear advantage of one over the other.

### Prolonged pH or pH-impedance monitoring

Prolonged pH or pH-impedance monitoring are tests known as the “gold standard” for the diagnosis of GERD, but they are not important in patients with LHH. These tests should be requested in order to decide about having surgery when there is doubt whether the patient’s symptoms or main complaint is related to gastroesophageal reflux^15^.

### Recommendations

Both upper digestive endoscopy and upper GI series are essential for the diagnosis and classification of LHH and should be requested for all patients with this type of hernia, being complementary to each other.

A CT scan helps identify multivisceral herniations (type IV) and is recommended for more complex hernias.

Esophageal manometry is recommended for patients complaining of dysphagia or when a fundoplication is planned.

A prolonged pH-monitoring study is unnecessary in most cases.

## CONSIDERATIONS ON SURGICAL TECHNIQUE

### Hernia sac management

- Finding the appropriate dissection plan and removing the hernia sac from the mediastinum is an important surgical step.
- Partial resection of the hernia sac with complete disconnection from the crura may be an alternative, especially in redos.

The hernia sac is of main importance in LHH surgical treatment. Often, the viscera, in particular the stomach, and EGJ cannot be reduced by traction back to the abdominal cavity because the entire posterior part of the sac covers the esophagus and is adhered to it.

The recommended surgical technique for LHH should be finding a plan for dissection between the hernia sac and the mediastinal structures and pleura, which is practically avascular, completely reducing the hernia sac to the abdominal cavity, and thereby, the EGJ and stomach would return to their anatomical position^16,51^.

In recurrent LHH, this correct plane is more fibrotic and adherent to the pleura, leading to higher risks of pleural injury. In the event of a capnothorax, within a few moments, chest and abdominal pressures are equalized and surgery can be continued with minor adjustments to ventilatory parameters by the anesthesiologist, associated with a reduction of the pneumoperitoneum pressure if needed.

It is strongly recommended that both vagal trunks be identified and preserved. In LHH, the posterior vagus nerve may be further away from the esophagus, increasing the risk of its injury.

After complete reduction of the hernia sac, it can be removed or transected to adequately expose the Angle of His, thus establishing an anatomical landmark for the EGJ to make sure an adequate length of the abdominal esophagus was achieved.

### Recommendations

The hernia sac must always be completely disconnected from the hiatus. Complete resection is optional.

It is strongly recommended to actively search and identify the anterior and posterior vagal trunks, due to a higher risk of iatrogenic injury.

### How cranial should the thoracic esophagus be dissected?

- Achieving an adequate abdominal length is of main importance on the surgical treatment of LHH.
- Mediastinal dissection must be as cranial as possible to reduce axial tension (cranial traction) so the esophagus maintains its abdominal position at the end of surgery, without traction.

The existence of an intra-abdominal segment of the esophagus is a natural antireflux mechanism since abdominal positive pressure collapses the esophageal walls by external pressure. Also, for decades, it has been demonstrated that reflux control is directly related to the length of the abdominal segment of the esophagus^15^. Thus, one of the objectives of antireflux surgery is to restore an adequate abdominal esophageal length, especially in patients with hiatal hernia. In addition, inadequate length of intra-abdominal esophagus is associated with the recurrence of hernias.

Restoration of abdominal esophagus length is achieved through circular dissection of the esophagus in the cranial direction. Therefore, the thoracic esophagus should be dissected to a sufficient extent to obtain satisfactory abdominal esophageal length^17,21^.

There are no studies comparing the benefits of different final lengths of the abdominal esophagus, and the *recommendation of experts* is to obtain 3 cm of extension, without caudal traction^17,41^.

In some cases, even after extensive dissection, adequate abdominal esophageal length may not be obtained, defining the so-called “short esophagus”. In such situations, other measures should be taken and will be discussed later on.

### Recommendations

The thoracic esophagus should be dissected in an extension sufficient to obtain 3 cm of abdominal esophagus, without traction.

### Can adjuvant techniques improve the results?

Axial tension from the upward traction forces of the esophagus may be involved in recurrence. Some techniques are described to try to reduce it.

#### Collis gastroplasty

Collis gastroplasty is a technical surgical step used to reduce axial tension when at least 3 cm of abdominal esophagus is not obtained after extensive mediastinal dissection.

Although efficient and potentially reducing hernia recurrences, this technique has some drawbacks.

Firstly, the morbidity of the operation increases, and fistula is a rare but possible occurrence. Secondly, the longer the stomach segment required in gastroplasty, the greater the number of parietal cells above the fundoplication, leading to pathological reflux in up to 50% of cases. Another concern is the presence of a gastric segment without peristalsis above the fundoplication, which may lead to dysphagia^23,48^.

Despite these potential problems, the quality of life reported in patients undergoing this technique is similar to those undergoing traditional fundoplication^33^.

#### Vagotomy

Truncal vagotomy was described by Oeschlager et al.^37^ as a simple maneuver to obtain EGJ reduction, and thus, avoid Collis gastroplasty. This is a solitary study, showing that when EGJ was not reduced to more than 2 cm in the abdomen without tension, performing truncal vagotomy led to achieving adequate abdominal length without the association of potential known adverse effects related to this maneuver (delayed gastric emptying or dumping).

With a scarcity of data in the literature, this should be an operation used with extreme caution since it is irreversible and has potential adverse effects.

#### Gastropexy or gastrostomy

Gastropexy is an attempt to fix the stomach in the abdominal cavity. Some forms have been proposed: fixation to the crura, Hill’s gastropexy (where the small curvature is fixed to the arcuate ligament), anterior gastropexy with nonabsorbable sutures, and finally, fixation by gastrostomy.

All these techniques of anterior gastropexies are described in the literature and presented as case series. There is insufficient evidence for a definitive conclusion. However, the frequency of recurrences described in those series reaches 15%, which is relatively low^1,3,39^.

### Recommendations

Collis gastroplasty should be part of the tactical armamentarium of the surgeon who deals with complex cases of hiatal hernia, but its use should be restricted to exceptional situations where it is not possible to reduce the EGJ to the abdominal cavity.

Different forms of gastropexy can be used, given the low rates of complications reported, but they do not serve as substitutes for adequate mobilization and reduction of the EGJ to the abdominal cavity.

### How should the hiatus be closed?

- Adequate hiatal closure around the esophagus is of main importance in the proper treatment of LHH.
- There is consensus in the literature that nonabsorbable sutures must be used, but suture technique (simple stitches, figure-of-eight, mattress, or continuous suture) and suture type (multifilament, monofilament, or barbed) are still the surgeon’s preference.

Axial and radial tensions, to which EGJ and diaphragmatic pillars are submitted, contribute to the high rate of recurrence seen in LHH. In this topic, we are interested in the radial tension on the crura.

Recent literature on the subject demonstrates that radial tension is proportionally related to the width and the area of the hiatus, and that the surgeon’s clinical impression does not always correspond to the tension objectively measured in the pillars. In addition, the shape of the hiatus (slit, drop, D, or oval) is also related to the closing tension of the pillars, which are larger in the D and oval configurations^4,34^.

In large, D-shaped or oval hiatus, when the tension is high, lateral sutures approaching the left crus to itself (at the 2 o’clock position, in analogy) may be necessary to adequate closure around the esophagus.

Regarding the closure itself, nonabsorbable sutures should be used, with no difference between continuous suture, separate or simple stitches, in X or U. The use of Teflon pledgets is popular among many services, but to this day has not proven to be beneficial in this scenario, prevailing here the preference of the surgeon.

Usually, the hiatal closure begins posteriorly, where the radial tension is lower and continues anteriorly. Excessive anterior angulation of the esophagus may cause dysphagia and should be avoided. Placing sutures anterior to the esophagus may be performed when necessary without compromising the efficiency of the repair.

In very large defects, some experts perform diaphragmatic relaxing incisions. By making an opening, on the right or left diaphragm, a reduction of the tension on the crural sutures is achieved. It is reported a reduction of 46% in the tension with relaxing incision alone, and a 56% reduction when associated with pleurotomy^4^. Such an incision produces a weak area at the incision site and must be fixed with an inlay, nonbsorbable mesh, placed away from the esophagus or fundoplication.

One large series describes the use of the relaxing incision in 11% of paraesophageal hernia cases (in the so-called more severe cases of suture tension). With this tactic, authors report recurrence similar to that observed in cases of lower severity. They draw attention to the importance of associating a prosthesis to cover the defect created by the incision. Although they did not report complications related to the prosthesis, there is a risk of iatrogenic injury due to its fixation next to the vena cava or thoracic duct, conditioning the use of this tactic to surgeons with high affinity with the technique^12^.

Another tactic maneuver described to help complete hiatal closure around the esophagus is the pleurotomy. The intentional creation of a capnothorax (usually on the left side), results in a “flattening” of the diaphragm, which reduces the tension at the hiatal sutures^4^.

### Recommendations

Complete closure of the hiatus around the esophagus with nonabsorbable sutures must be obtained. An intraesophageal bougie should be used to avoid tight closure or anterior angulation of the esophagus.

Regarding adjuvant maneuvers to help hiatal closure, such as pleurotomy and relaxing incisions, the lack of consistent studies suggests that their use should be based on a case-by-case evaluation, highly depending on the surgeon’s expertise.

### Can the use of mesh improve results?

- The use of mesh as a method to reduce recurrences of LHH is based on the success they had in other abdominal wall hernias but this positive effect is still a matter of great debate among specialists.
- The use of nonabsorbable prostheses has a good effect on preventing anatomical recurrence in short-term follow-up but without long-term evidence. Its use can potentially cause severe complications, requiring complex surgery to treat, associated with high morbidity and mortality rates.
- Absorbable meshes can reduce short- and medium-term recurrences, but the long-term follow-up is still under investigation.

Prospective and randomized studies in the literature have conflicting results on reducing recurrences using prostheses to reinforce hiatoplasty. A major limitation is that the few randomized studies analyzed different types of meshes, varying in relation to the material (polypropylene, PTFE, biological, absorbable synthetic), shape (U-, C-, rectangular, circumferential), size (1x3 cm, 3x4 cm), fixation methods (points, staples, glues), and application position (anterior or posterior to the esophagus), in addition to having different definitions as to what large hernias would be^11,12,30,40^.

The use of permanent prosthesis is described to be associated with severe complications: during the surgery, when there are accidents with tacks fixation; and later on the follow-up, such as intraluminal erosion (esophagus or stomach) and stenosis at the EGJ due to retraction and dense fibrosis, which usually require complex visceral resections to treat^5,44^.

These risks, despite being isolated case reports, led to a reluctance to apply a permanent prosthesis on the hiatus and the search for biological prostheses, which would be absorbed after a certain time. However, a randomized controlled trial showed long-term recurrence rates similar in mesh and non-mesh groups^35,36^.

Recently, absorbable synthetic meshes were introduced, aiming to induce local proliferation of fibroblasts, favoring the formation of a more resistant scar as well as producing a local inflammatory reaction that would cause greater fixation of the esophagus/fundoplication complex in the hiatus region^40^.

A recent meta-analysis, comparing synthetic absorbable meshes to no mesh, reported a significantly lower recurrence rate than the non-mesh group (8 vs. 18%; 95%CI 0.08−0.17). Long-term results of these types of mesh are still unknown^11^.

### Recommendations

Based on recent literature, there are no data that can contraindicate or strongly recommend the use of prosthesis as an absolute way to reduce recurrence of LHH.

The use of prosthesis to reinforce hiatoplasty remains a surgeon’s personal choice, based on experience, always considering the potential risks of complications in the short and long term.

We recommend that, if a mesh is to be applied, it should be resorbable and fixed with suture or glue.

Mesh must not be placed without a complete approximation of the hiatus around the esophagus. There is no indication of using mesh as a bridge or inlay repair.

### Is fundoplication necessary?

- Fundoplication is recommended by the majority of experts during the surgical management of LHH.
- Fundoplication lowers the occurrence of GERD, which is very common after a hernia correction.
- Despite lacking strong evidence in the literature, it is suggested that fundoplication should also lower the risk of LHH recurrence.

The addition of a fundoplication to LHH repair is controversial. It prevents gastroesophageal reflux disease that may occur even if it was not present previously due to extensive dissection of the EGJ and disruption of natural antireflux mechanisms. Moreover, the wrap may help keep the stomach in the abdominal position.

On the other hand, some authors recommend only selective performance of fundoplication, that is, only in those patients with evidence of reflux, due to the fact that fundoplication may be associated with postoperative complications such as dysphagia and gas bloat.

In a recent study, 60 patients who underwent surgery for LHH were divided into 35 patients with fundoplication and 25 without. In patients where fundoplication was not performed, despite not having reflux previously, there was a 28% incidence of esophagitis and 39% of abnormal acid exposure^19^.

Upon decided that a fundoplication will be part of the procedure, whether it should be a total or partial fundoplication is still a matter of debate. Each one has its own pros and cons, but the general consensus is that if the aim of the fundoplication is to prevent long-term reflux, it favors a total fundoplication. If the main goal is preventing gas bloat and dysphagia, a partial fundoplication should be the choice.

### Recommendations

A fundoplication should be added routinely to the LHH repair. Besides restoring the anatomy after extended EGJ dissection, it helps keep the stomach in the abdominal situation, preventing hiatal hernia and GERD recurrence.

We recommend a total fundoplication, but a partial may also be used specially in the setting of old age, dysphagia, or dysmotility.

### Robotic platform

Robotics is a stable platform with many ergonomic advantages for the surgeon. For LHH repair, the stability of optics and the ability of the robotic arms to work parallel to the esophagus within the restricted space of the mediastinum may help achieve an easier and higher dissection of the proximal esophagus.

In longer surgeries, specially LHH in a redo situation, the comfort delivered to the surgeon in the console favors better surgical technique.

There are not yet high-quality prospective studies, clinical trials, or meta-analyses of results to compare robotic LHH repair to laparoscopy. A low-quality systematic review disclosed the similarity of results^49^. A populational study with almost 170,000 individuals, however, showed a higher index of complications in robotic surgery, especially pulmonary and esophageal perforations, even when matched for the surgeon´s expertise^50^.

Some studies evaluated robotic surgery in special situations. The repair of recurrent HH showed similar results to the index operation in 51 patients^42^. Robotic surgery was superior in emergency cases according to a multicenter study; however, the procedure was performed only by the most experienced teams in the emergent setting^22^.

It is undeniable that the robotic platform is a huge advance in technology applied to minimally invasive surgery, adding ergonomics, stability of the camera, and precision of dissection. However, it is still waiting for consistent data to conclude whether it contributes to better results than laparoscopic surgery, both conducted by teams with experience in complex LHH surgeries.

### Recommendations

Robotic platforms may add stability to dissection and ergonomic advantages in LHH surgeries. There are no clear complications or advantages in outcomes when compared to laparoscopic approach when both are performed by experienced surgeons.

## SPECIAL SITUATIONS

Specific clinical scenarios may demand a different approach when managing LHH.

### Acute complications (emergency scenario)

Emergent situations may occur in the LHH setting. Upper digestive hemorrhage is a rare but possible situation. Fortunately, it is generally controlled by clinical and endoscopic methods^25^. The most common and morbid condition is obstruction usually followed by ischemia of the stomach which comprises 98% of emergent operations cases. Almost 30% of patients with acute volvulus may present with ischemia leading to a 50% mortality rate^6^. Obstruction is caused by gastric volvulus. Classically, it is clinically represented by the Borchardt triad: thoracic (or upper abdominal) pain, impossibility to vomit, and inability to pass a nasogastric tube^6^.

An upper digestive endoscopy must be the initial approach in emergency to try emptying the stomach and locating a nasogastric tube for decompression. These acts may prevent ischemia and eventually devolvulate the stomach or postpone surgical therapy to allow clinical condition improvement.

Emergency surgery becomes the only option in cases of failure of endoscopic decompression or signs of ischemia and/or sepsis. Complex operations may be anticipated and gastric resection may be necessary. HH should be treated and an alternative feeding route provided. Access may be through laparotomy or a minimally invasive procedure depending on the expertise of the team. A recent study showed that matched groups have double the risk for severe complications and three times more mortality in emergency operation even if age and comorbidities are considered^46^.

### Obesity

Obesity is a risk factor for HH. One third of the obese population may have HH and 5% will present LHH^8^. Moreover, obesity is linked to higher rates of recurrence^29,31^.

Obese patients, as well as patients with increased intra-abdominal pressure due to other causes such as abdominoplasty, have worse long-term results following HH repairs and should be also managed with caution in events of LHH.

In cases of obesity grades I and II without comorbidities, weight loss guidance before the surgical procedure is common in clinical practice, despite presenting irregular results. In morbidly obese patients, there is already consensus that a bariatric procedure should be added to the hernia correction. Roux-en-y gastric bypass is the recommended technique because it presents better results in treating reflux, with good weight loss, control of comorbidities, and reduction of LHH recurrences^43^.

Sleeve gastrectomy is associated with the development of postoperative reflux, presents a greater chance of herniation, and thus, should be avoided.

### Old age

LHH are frequently found in the elderly with an average age between 65 and 75 years. Safety, quality of life, and recurrence do not seem to be affected by age, even considering a group of octogenarians. Time of hospitalization, need for intensive care and mechanical ventilation were, however, longer in the oldest^38^.

### Recommendations

Endoscopic therapy should always be tried as a first tentative to decompress the stomach and locate a nasogastric tube at least to prepare the patient for a definite surgical treatment in better clinical conditions in the emergency setting. Cases of ischemia and sepsis should be treated with surgical therapy. Minimally invasive procedures are the preferential route, when possible, and sepsis control and a feeding route are mandatory.

A bariatric procedure should be added in the morbidly obese. Roux-en-y gastric bypass is the procedure of choice.

Treatment of LHH in elderly should be performed in symptomatic patients electively with results and similar safety to non-elderly patients. Technical alternatives such as partial fundoplication and anterior gastropexy can be considered.
